# Supplementary material for: Midzone bundles of the mammalian anaphase spindle are mechanically coupled both locally and globally
Source: bioRxiv. 2026 Apr 10:2026.04.08.716168. Preprint. [Version 1] doi: 10.64898/2026.04.08.716168 (PMC13081819; doi:10.64898/2026.04.08.716168)

bioRxiv preprint doi: https://doi.org/10.64898/2026.04.08.716168; this version posted April 10, 2026. The copyright holder for this preprint (which was not certified by peer review) is the author/funder, who has granted bioRxiv a license to display the preprint in perpetuity. It is made available under aCC-BY 4.0 International license.

Supplementary and video legends

S1. **Midzone bundle lateral movement additional analysis. (A)** Natural log transformation of data shown in Fig. 2 C and Fig. 2 F with simple linear regression plotted. Slopes are not different ($p = 0.93$, analysis of covariance).

Video 1. **Movie of representative on-target fast manipulation of the anaphase spindle.** Fluorescence confocal microscopy movie showing microneedle (BSA Alexa Fluor 647, magenta) manipulation of PtK2 GFP-α-tubulin (green) anaphase midzone bundle. Time (in seconds) is shown where time = 0 is the start of manipulation (7fps). Scale bar = 5 μm. See also Fig. 1D.

Video 2. **Movie of representative off-target fast control manipulation next to the anaphase spindle.** Fluorescence confocal microscopy movie showing microneedle (BSA Alexa Fluor 647, magenta) control manipulation just outside the spindle region in a PtK2 GFP-α-tubulin (green) cell in anaphase. Time (in seconds) is shown where time = 0 is the start of manipulation (7fps). Scale bar = 5 μm. See also Fig. 1E.

Video 3. **Movie of a representative unperturbed anaphase cell.** Fluorescence confocal microscopy movie showing an unperturbed PtK2 GFP-α-tubulin (green) anaphase cell. Time (in seconds) is shown where time = 0 is the start of imaging (7fps). Scale bar = 5 μm. See also Fig. 1F.

Video 4. **Movie of a representative on-target slow manipulation showing lateral midzone bundle coupling in the anaphase spindle.** Fluorescence confocal microscopy movie showing microneedle (BSA Alexa Fluor 647, magenta) manipulation of PtK2 GFP-α-tubulin (green) anaphase midzone bundle and concordant neighbor bundle movement. Time (in seconds) is shown where time = 0 is the start of manipulation (7fps). Scale bar = 5 μm. See also Fig. 2B.

bioRxiv preprint doi: https://doi.org/10.64898/2026.04.08.716168; this version posted April 10, 2026. The copyright holder for this preprint (which was not certified by peer review) is the author/funder, who has granted bioRxiv a license to display the preprint in perpetuity. It is made available under aCC-BY 4.0 International license.

Video 5. **Movie of a representative on-target fast manipulation of the anaphase spindle showing spindle length shrinkage upon force application.** Fluorescence confocal microscopy movie showing fast microneedle (BSA Alexa Fluor 647, magenta) manipulation of PtK2 GFP-α-tubulin (green) anaphase midzone bundle and spindle length shrinkage. White bars mark pre-pull spindle length. Frames during manipulation duplicated for emphasis. Time (in seconds) is shown where time = 0 is the start of manipulation (7fps). Yellow scale bar = 5 μm. See also Fig. 3C.

Video 6. **Movie of a representative slow microneedle manipulation of the anaphase spindle.** Fluorescence confocal microscopy movie showing slow microneedle (BSA Alexa Fluor 647, magenta) manipulation of PtK2 GFP-α-tubulin (green) anaphase midzone bundle. Frames during manipulation duplicated for emphasis. Time (in seconds) is shown where time = 0 is the start of manipulation (7fps). Yellow scale bar = 5 μm. See also Fig. 4A.

Video 7. **Movie of representative on-target fast manipulation of the anaphase spindle with PRC1 siRNA.** Fluorescence confocal microscopy movie showing microneedle (BSA Alexa Fluor 647, magenta) manipulation of PtK2 GFP-α-tubulin (green) anaphase cell treated with PRC1 siRNA. Frames during manipulation duplicated for emphasis. Time (in seconds) is shown where time = 0 is the start of manipulation (7fps). Yellow scale bar = 5 μm. See also Fig. 5D.

Figure S1: Fast and slow lateral connections show similar trends across space

A

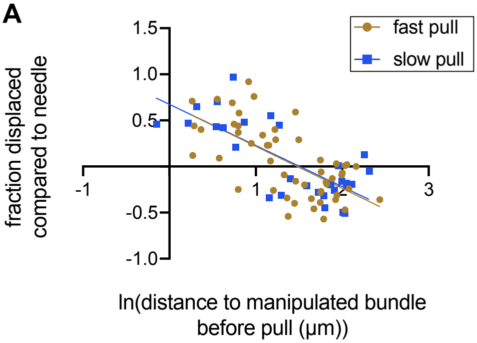

Supplement: Supplement 8 — S1. Midzone bundle lateral movement additional analysis. (A) Natural log transformation of data shown in Fig. 2 C and Fig. 2 F with simple linear regression plotted. Slopes are not different (p = 0.93, analysis of covariance). [file NIHPP2026.04.08.716168v1-supplement-8.pdf]
